# Microstructure and Characteristic of BiVO_4_ Prepared under Different pH Values: Photocatalytic Efficiency and Antibacterial Activity

**DOI:** 10.3390/ma9030129

**Published:** 2016-02-25

**Authors:** Zhengyao Qu, Peng Liu, Xiaoyu Yang, Fazhou Wang, Wenqin Zhang, Chenggang Fei

**Affiliations:** 1 State Key Laboratory of Silicate Building Materials, School of Materials Science and Technology, Wuhan University of Technology, Wuhan 430070, China; 2 School of Chemistry, Chemical Engineering and Life Science, Wuhan University of Technology, Wuhan 430070, China; 3 State Key Laboratory of Advanced Technology for Materials Synthesis and Processing, Wuhan University of Technology, Wuhan 430070, China

**Keywords:** BiVO_4_, monoclinic scheelite structure, photocatalytic efficiency, *E. coli*, envelop

## Abstract

In the present study, BiVO_4_ sample was prepared under different pH 0.5–13 without capping agent. Different morphology characteristics were observed, such as sheet crystal structure, cross crystal structure and branching crystal structure. The mechanism of the formation of BiVO_4_ nanostructure was discussed. Under acid condition, sheet crystal structure was obtained. The phenomenon could be attributed to polymerization of vanadate in the presence of H^+^. In the weak alkaline solution, across structure and branching type morphology was obtained. The photocatalytic efficiency for the samples ranked as pH 5 > pH 3 > pH 7 > pH 9 > pH 1 > pH 11 > pH 13 > blank, which is in good agreement with X-ray diffraction (XRD) result. *E. coli* envelop was damaged in the presence of BiVO_4_ under visible light. The protrusion on envelop was diminished by BiVO_4_. Attenuated Total Reflection Fourier transformed Infrared Spectroscopy (ATR-FTIR) results suggested the intensity was weakened for the amide, phosphoric, –COO^−^ group and C-H bond in lipopolysaccharides (LPS), peptidoglycan and periplasm molecules.

## 1. Introduction

Degradation of organic pollutants or hydrogen production from water splitting over a semiconductor become one of the most important means for further maximizing the efficiency of solar energy [1,2,3,4]. Titanium dioxide (TiO_2_) is the most popular material used in heterogeneous photocatalysis for its excellent properties, such as high stability, chemical inertness, non-toxicity, and low cost [1,3,5,6]. However, the band gap of anatase (3.2 eV) is not handled easily for solar applications, which limits wide application in visible range [6,7,8].

To improve the photocatalysis efficiency under visible light, many methods have attempted to decrease the band gap energy [9,10,11,12]. A series of new visible light photocatalysts were designed and prepared, such as Bi_2_WO_6_ [13,14], BiVO_4_ [15], InVO_4_ [16], Sr_2_Nb_2_O_7_ [17], and Sr_2_Ta_2_O_7_ [18], so as to make full use of the convenient solar energy.

Among them, BiVO_4_ has become a new promising candidate material. There are three crystalline phases reported for synthetic BiVO_4_, namely, a monoclinic scheelite, a tetragonal zircon and a tetragonal scheelite structure. Among these phase structures, the monoclinic scheelite structure of BiVO_4_ possesses the best photocatalytic performance under visible-light irradiation due to its relatively narrow band gap of 2.4 eV, compared to the two tetragonal phases with the band gap energy of 3.1 eV [19,20,21]. Therefore, the preparation of monoclinic BiVO_4_ phase is greatly important to make use of sunlight to degrade the organic pollutants.

Researchers have used the precipitation, sol–gel and hydrothermal method to prepare BiVO_4_ [22,23,24]. Some factors limited the application of precipitation and sol–gel method due to raw material cost, and some extreme reaction condition [23]. The hydrothermal method is widely used to prepare BiVO_4_ with the high photocatalytic activity with narrow band gap. The effect of temperature, reaction time on the preparation of BiVO_4_ was analyzed and discussed. The pH values were also an important factor for the formation of BiVO_4_ [25]. 

The photocatalysts is expected to inhibit growth of bacteria and be utilized in water treatment plants [26,27,28]. The mechanism for its inhibitory effect on bacteria is based on the active substance produced during the photo-catalytic process. The free radical produced during the process could damage many organic covalent bonds, such as C-C, C-H, C-N, C-O, and H-O. 

In the present work, monoclinic BiVO_4_ (m-BiVO_4)_ was prepared by hydrothermal method under different pH values (0.5–14). We used the mixture of BiCl_3_ and NH_4_VO_3_ as precursor without capping agent and the procedure avoided microwave reaction and anneal, which is different with previous reports [29,30]. The alteration of morphology and crystal of BiVO_4_ with pH values was observed and discussed. The photocatalytic efficiency of BiVO_4_ was measured using Rhodamine B as a target. Optimal pH values and morphology were determined according to photocatalytic activities. The damage to *E. coli* envelop caused by BiVO_4_ under visible light was observed and measured. Rhodamine B is a chemical compound containing amino-group, carboxyl group, methyl group and phenyl group. Most of them are similar to the groups of biomolecules on cell envelop. Rhodamine B is also used extensively in biotechnology applications such as fluorescence microscopy due to its ability to combine with biomolecules. Both degradation of Rhodamine B and damage of *E. coli* envelop can show photocatalytic activity of BiVO_4_ under visible light. 

## 2. Materials and Methods

### 2.1. Preparation of BiVO_4_

BiCl_3_ (99%), NH_4_VO_3_ (99%), purchased from Aladdin and all chemicals were analytical grade and were used directly without further purification. Other chemicals are analytical grade. The BiVO_4_ photocatalysts were prepared by a hydrothermal method without template or organic surfactant. In a typical synthesis process, stoichiometric amounts of BiCl_3_ (1 mmol) and NH_4_VO_3_ (1 mmol) were dissolved in 80 ml of distilled water and an orange suspension was formed under mild stir. The pH value of the orange suspension is 2.30. Then the pH value was adjusted by HCl or NaOH to 0.5, 1, 3, 5, 7, 9, 11, 13, respectively. After stirring for 0.5 h, the obtained mixture was autoclaved into a Teflon-lined autoclave at a temperature of 180 °C for 24 h and then naturally cooled to room temperature, for approximately 3 h. The sample with yellow color was collected, washed with de-ionized water and alcohol three times respectively and dried at 80 °C in a vacuum for 24 h.

### 2.2. Characterization

X-ray diffraction (XRD) patterns of the BiVO_4_ prepared under different pH value were recorded on a D/max-γA X-ray diffractometer (Rigaku, Tokyo, Japan) equipped with graphite monochromatized Cu-Kαradiation (λ = 1.54178 Å). Field emission scanning electron microscope (FESEM, JEOL JSM 6700F field emission JEOL, Tokyo, Japan) is used to analyze the morphologies of the particles and bacteria cells after disinfection. UV-Vis diffuse reflectance spectra were measured by a Perkin Elmer Lambda 750 Spectrometer (Perkin Elmer, Waltham, MA, USA). The concentration of RhB during the degradation was recorded by colorimetry with a UV-vis spectrometer (721 Shanghai Lengguang Tech., Shanghai, China) at λ_max_ = 553 nm. Attenuated total reflectance Fourier transform infrared spectroscopy (ATR-FTIR, Nicolet 6700* Thermo Nicolet, Waltham, MA, USA) is chosen to observe functional group changes of the envelope of *E. coli*.

### 2.3. Photocatalytic Degradation of Rhodamine B

The photocatalytic activities of the BiVO_4_ were evaluated by degradation of Rhodamine B in solution under visible light from a 300W Xe lamp (CERMAX LX−300; ILC Technology, Fremont, CA, USA) equipped with cutoff filter L42 (Hoya Optics) and a water filter. The photocatalyst (0.01 g) was added into 50 mL RhB aqueous solution (10 mg L^−1^) in a beaker at room temperature under air. Before light was turned on, the solution was continuously stirred for 30 min in dark condition to ensure the establishment of the adsorption–desorption equilibrium. After 30 min, 5 mL of suspension was taken from the mixture and centrifuged at 4000 rpm to separate the photocatalyst particles. Subsequently, samples were taken at 30 min intervals.

### 2.4. SEM Observation of E. coli

All glasses and materials were sterilized at 120 °C for 40 min in an autoclave. When *E. coli* was grown to primary-log phase at 37 °C in a peptone culture, two groups of bacteria suspension were centrifuged at 4000 rpm for 5 min. The precipitate was resuspended in PBS buffer. In the same way, the bacteria were washed twice to thoroughly remove the culture medium. The cells were treated as follows. A (the control): the native cells in PBS with shaking for 1 h; B: the cells in PBS in the presence of BiVO_4_ which is prepared under pH = 5 under iodine tungsten lamp with a UV filter (300 W, FoShan lighting). After photo-catalysis, the samples were processed before FESEM obervation. Glutaraldehyde was used to fix the protein (or lipid) in cells. Then the cells were dehydrated in a series of increasing concentration of ethanol (50, 60, 70, 80, 90, and 100%). Subsequently, the cells were fixed and dehydrated. They were observed by SEM (HITACHI, S-4800).

### 2.5. Changes of Molecules on E. coli envolop Observed by ATR-FTIR 

When *E. coli* was grown to primary-log phase at 37 °C in a peptone culture, four groups (labeled A, B, C and D, respectively) of bacteria suspension were washed as described as section 2.4. The four respective groups of cells were treated by photocatalysis in the presence of BiVO_4_ with pH 5 for different time, as follows. A (the control): the native cells in PBS with shaking for 1 h; B: the cells in PBS which is under iodine tungsten lamp with a UV filter (300 W, FoShan lighting, Foshan, China). The ATR-FTIR spectra of seven groups of *E. coli* were measured by FT-IR spectroscopy. Then the samples were collected and dried in a vacuum chamber. Spectra were the results of 64 scans with a resolution of 4 cm^-1^ in the spectra range 4000–600 cm^−1^.

## 3. Results and Discussion

### 3.1. XRD Patterns of the BiVO_4_ Prepared under Different pH Value

**Figure 1:**
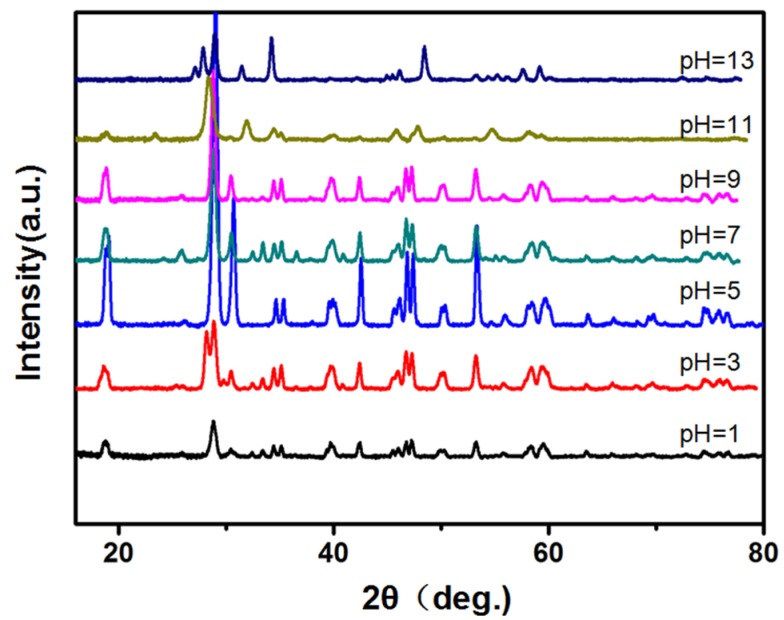
X-ray diffraction (XRD) patterns of the BiVO_4_ samples prepared at different hydrothermal pH values.

XRD patterns of BiVO_4_ prepared under different pH 1–13 value without capping agent were shown in Figure 1. The XRD patterns are in good agreement with the standard Joint Committee on Powder Diffraction Standards (JCPDS) card No. 14-0688, which is assigned to monoclinic BiVO_4_. The splitting of the peaks at 2θ (18.5°, 35°, and 46°), which is characteristic of the monoclinic structure of BiVO_4_ [21]. Three crystalline phases were reported for BiVO_4_, that is, a monoclinic scheelite, a tetragonal zircon and a tetragonal scheelite structure [15,16]. Among these phase structures, the monoclinic scheelite structure of BiVO_4_ possesses the best photocatalytic performance under visible-light irradiation. The matching degree of XRD patterns with JCPDS for BiVO_4_ samples can rank as pH 5 > pH 3 > pH 7 > pH 9 > pH 1 > pH 11 > pH 13 > blank. There is no impurity peaks, indicating that pH (≤9) is suitable for the formation of phase-pure monoclinic BiVO_4_ hydrothermally. The intensity of the peaks increased from pH 1 to 5 and then decreased from pH 5 to 13, especially at 2θ = 18.5° (110, 011 face), 27.5° (121, 040 face). This indicates that the BiVO_4_ sample prepared under pH 5 might possess highest crystallization degree. BiVO_4_-1 sample might have anisotropic growth along the (010) plane.

### 3.2. Morphology Variation of BiVO_4_ Prepared under Different pH

**Figure 2:**
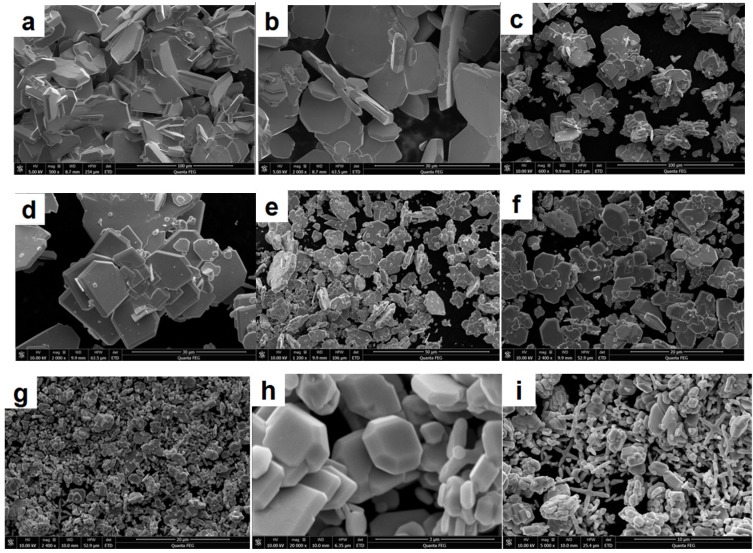

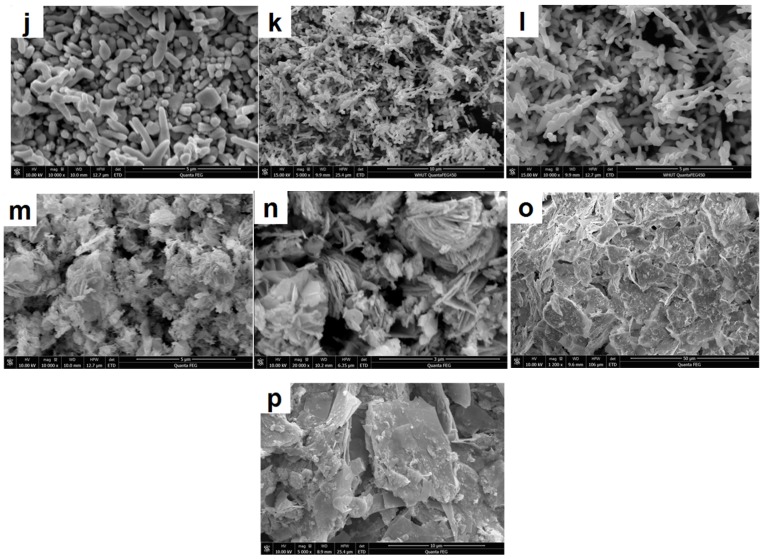
Morphology of BiVO_4_ prepared under pH = 0.5 (**a**;**b**), pH =1 (**c**;**d**), pH = 3 (**e**;**f**), pH = 5 (**g**;**h**), pH = 7(**i**;**j**), pH = 9 (**k**;**l**), pH = 11 (**m**;**n**), pH = 13 (**o**;**p**).

BiVO_4_ nanoarchitectures were synthesized by the reaction between Bi^3+^ and VO_4_^3−^ ions by a hydrothermal method at 180 °C without capping agent. Figure 2 has shown the morphology of BiVO_4_ prepared under different pH values from 0.5 to 13. Under acid condition, sheet crystal structure was observed. With concentration of H^+^ increased in solution, the sheet was formed larger and larger. The length even reached 30 µm under pH 0.5. The variation of length and thickness with pH value was shown in Table 1.

Generally, the free VO_4_^3−^ only exists in the strong alkaline solution. With addition of H^+^, poly-vanadate will be produced due to polymerization with different degree. With the increase of the concentration of H^+^, the oxygen atom in the vanadate is taken by H^+^ gradually. With the decrease of pH value, the degree of polymerization increases further as follows:

$$\begin{array}{l} 2{\mathrm{VO}}_{4}^{3-}+2H^{+}⇄2{\mathrm{HVO}}_{4}^{2-}⇄V_{2}O_{7}^{4-}+H_{2}O\\ 3V_{2}O_{7}^{4-}+6H^{+}⇄2V_{3}O_{9}^{3-}+3H_{2}O\\ 10V_{3}O_{9}^{3-}+12H^{+}⇄3V_{10}O_{28}^{6-}+6H_{2}O\\ {\left[V_{10}O_{28}\right]}^{6-}+H^{+}⇄{\left[HV_{10}O_{28}\right]}^{5-}\\ {\left[HV_{10}O_{28}\right]}^{5-}+H^{+}⇄{\left[H_{2}V_{10}O_{28}\right]}^{4-} \end{array}$$

Therefore, under acid condition, it is easier to form BiVO_4_ of the sheet crystal structure. In the strong acid solution (pH ≤ 1), however, Bi^3+^ was adsorbed quickly on the surface of poly-vanadate. According to the Gibbs-Curie-Wulff theorem, the growth rate is adverse to the atom density of the respective plane. BiVO_4_ sample with crystal form couldn’t be produced in strong acid solution. As shown in Figure 2g,h, BiVO_4_ sample with perfect crystal form was prepared under pH = 5. The FESEM image show that the crystal plane is very smoothing and clear, which is in agreement with XRD results. The as-obtained BiVO_4_ crystals possessed perfect octahedron and decahedron morphologies with sharp corners and well-defined edges. Li has reported experimental evidence for the separation of electrons and holes between the {010} and {110} crystal facets of BiVO_4_ [31]. It is expected that the BiVO_4_ sample prepared under pH 5 might have the highest photo-catalytic efficiency.

Under pH 7 and 9, across structure and branching type morphology was observed, respectively. BiVO_4_ with across structure is not very popular in Figure 2i,j,k,l. BiVO_4_ with branching type morphology has a length of about 3 µm and 200 nm in diameter in Figure 2k,l, indicating a high yield of these three-dimensional structures. Branching type is supposed to form by the connection of starlike product under pH 9. In the alkaline solution, Bi^3+^ concentration is very low. The reaction between Bi^3+^ and VO_4_^3−^ produced the nanoparticles, which will assembly into a new across structure under a hydrothermal condition at 180 °C, then the across structure will form the branching type morphology. An increase in pH would result in a relatively high value of supersaturation of the solution. The crystals grew along the different directions at different growth rates. Due to high supersaturation of the solution, the growth of the crystal plane with the higher growth rate would slow down, while the growth of crystal plane with the lower growth rate would increase [29,30]. As a result, the crystal plane of the (121) facets became smaller while the crystal plane of the (040) facets became larger, resulting in the morphology change and disappearance of the apexes of octahedron crystals. With OH^−^ concentration increased further to pH 11 and 13, the hamorphous sheet structure appeared because the reaction between Bi^3+^ and VO_4_^3−^ hardly happened.

### 3.3. Degradation Performance of Rhodamine B 

**Figure 3:**
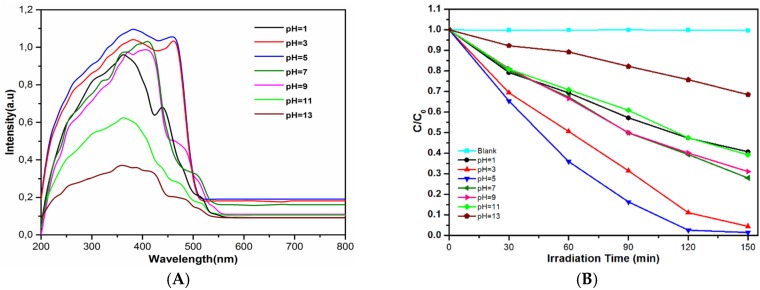
UV-vis diffuse reflectance absorption (**A**); and photocatalytic efficiency (**B**) of BiVO_4_ prepared under different pH values.

Researchers have reported UV-vis diffuse reflectance absorption spectra of BiVO_4_ [29,30,32]. The UV-vis diffuse reflectance absorption spectra of BiVO_4_ in our study were shown in Figure 3A. The absorption edge of the prepared BiVO_4_ is about at 530 nm. So the samples can absorb the visible light and the materials show photocatalytic activity in visible light region. Most of the samples showed absorption curve in the range of 420–500 nm. The sample prepared at pH 5 showed strongest absorption in the visible-light regions. The sample prepared at pH 11 and 13 showed weak absorption in the visible-light regions.

Figure 3B shows the photocatalytic degradation of RhB using the BiVO_4_ prepared under different pH. It can also be used as a test molecular for measuring the catalytic efficiency. In our experiment, the mixture contains 0.01 g photocatalyst and 50 mL RhB (10 mg L^−1^). The absorption of Rhodamine B on the material surface can be neglected because solution contains 0.02% material. In Figure 3, it can be seen that RhB were degraded gradually in the presence of catalysts under irradiation and control experiments (noted as blank) presents that in the absence of the lighting, RhB cannot be degraded by BiVO_4_ along. In addition, the most efficient catalyst to degrade RhB was BiVO_4_ which prepared under pH = 5. After 150 min, RhB molecules were decomposed completely. The photocatalytic efficiency ranked as pH 5 > pH 3 > pH 7 > pH 9 > pH 1 > pH 11 > pH 13 > blank. This result is in good agreement with XRD and SEM result. Perfect crystal in BiVO_4_ sample leads to high photocatalytic efficiency.

### 3.4. Outer Membrane Damage Observed by FESEM

**Figure 4:**
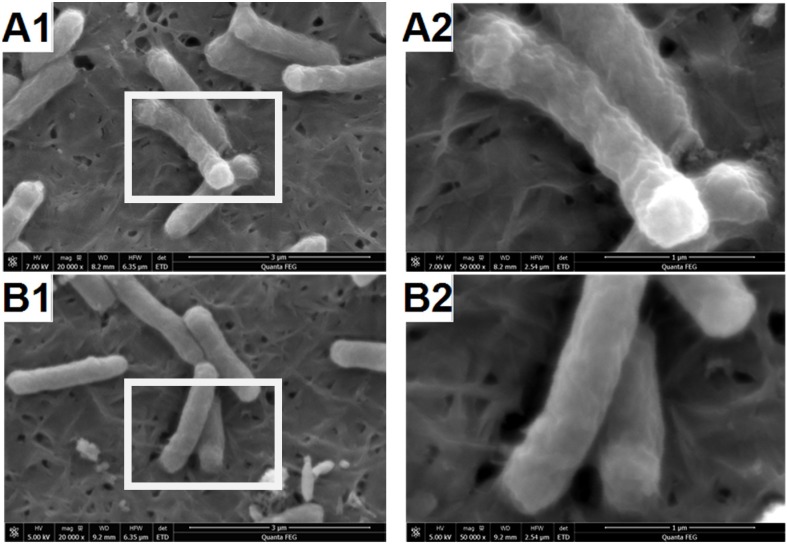
Field emission scanning electron microscope (FESEM) images with different magnification of bacteria before (**A1** and **A2**) and after (**B1** and **B2**) treatment of BiVO_4_ with illumination of visible light. The cells in image A2 and B2 was derived from image A1 and B1 separately with higher magnification.

**Figure 5:**
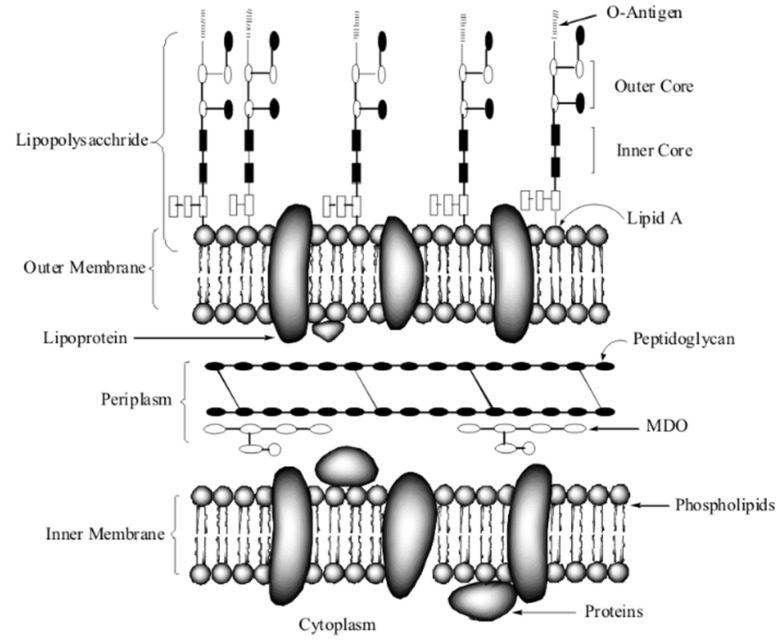
Schematic molecular representation of *E. coli* envelops.

In this study, the surface changes of *E. coli* cell were investigated by FESEM. The microstructures of the 2 samples were obtained, as shown in Figure 4. It is reported that the surface structure of the native *E. coli* was almost intact, with regular wrinkles at nano-level resolution [33]. In our images, the uniform protrusion was observed on the *E. coli* in Figure 4A1,B1. However, after reaction with BiVO_4_ the surface of cells became smoothing. The inner membrane of *E. coli* is composed of phospholipid chains and proteins, while the outer membrane consists of LPS, peptidoglycan and periplasm which was exposed to environment. Schematic molecular representation was shown in Figure 5. The observed protrusion was caused by LPS the tightly packing LPS patches. Under the visible light, BiVO_4_ produced electrons and holes between the {010} and {110} crystal facets. Both of them could react with LPS molecules. As a result, the damage caused the relaxing of LPS patches.

### 3.5. Functional Group Changes Observed by ATR-FTIR

**Figure 6:**
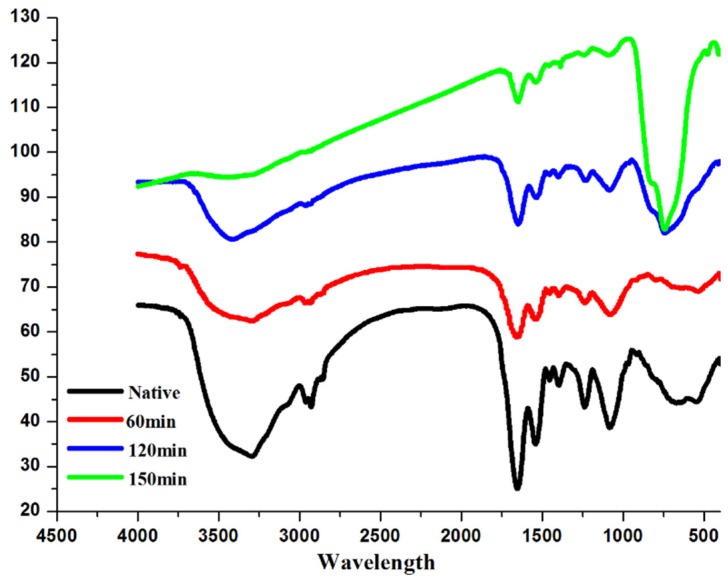
Attenuated total reflection fourier transformed infrared spectroscopy (ATR-FTIR) of *E. coli* envelop after treatment of BiVO_4_ with illumination of visible light.

To confirm the damage of *E. coli* envelope, the ATR-FTIR was measured after BiVO_4_ treatment under visible light. The envelope of *E. coli* is composed of LPS, a phospholipid layer, peptidoglycan (periplasm), as shown in Figure 5. The proteins and the glycoproteins were attached to the membrane. The ATR-FTIR spectra of the native *E. coli* are shown in Figure 6. According to the paper [34], the sharpest and most prominent peaks which belonged to the amide groups are 3301, 1658 and 1539 cm^−1^. As we know, the membrane is made of lipids and protein. Phospho-lipids, which form the bilayer structure, consist of a hydrophilic circular head and two hydrophobic fatty tails. The outer layer of the phospholipid bilayer is the hydrophilic end and the hydrophobic end is in between. Because the amide groups connected to the hydrophobic head are exposed to the environment, these groups showed the most significant peaks. Peaks at ~2962, 1082 were also prominent. These groups were attributed to υ_a_ (CH_2_), phosphoric acid asymmetric vibration of v (P = O), and the -C-O-C- in oligosaccharides which are bonded to proteins to form glycoproteins. Small peaks at ~2959, 2856 and 1457 cm^−1^ were due to C-H bond, 1042 cm^−1^ to –COO^−^ in hydrophobic glycerol end, 1237 cm^−1^ to symmetric vibration of PO_2_^-^ and 1151 cm^−1^ to v (C-O). The peak intensity decreases indicated the group on cell envelops was damaged. The results suggest the outer leaflet damage of amide groups in the hydrophobic end of the phospholipids. The asymmetric stretching mode ν_as_ (PO_2_^−^) of the phospholipid phospho-diester bond also decreased significantly because the outermost groups exposed to the irradiation were the easiest to be oxidized. The groups CH_2_ and CH_3_ deceased at a slower pace with a higher resistance to irradiation.

ATR-FTIR is reported to be a suitable technique to follow the structural changes of the *E. coli* cell membranes during photocatalysis [35]. Formation of the peroxidation products was confirmed due to the photocatalysis of *E. coli* cell envelope. Time dependent ATR-FTIR experiments provide the evidence for the changes in the *E. coli* cell wall membranes as the precursor events leading to bacterial lysis. The interactions of TiO_2_ with phospolipid bilayers found in cell membrane walls were also observed [36].

## 4. Conclusions

BiVO_4_ sample was prepared under different pH 0.5–13 without capping agent. XRD patterns confirmed the formation of BiVO_4._ Different morphology characteristics were observed, such as sheet crystal structure, cross crystal structure and branching crystal structure. Under acid condition, sheet crystal structure was observed. With increased concentration of H^+^ in solution, an increasingly large sheet was formed. The phenomenon could be attributed to polymerization of vanadate in the presence of the concentration of H^+^. In the weak alkaline solution, morphology was observed across structure and branching type. In the alkaline solution, Bi^3+^ concentration is very low. The reaction between Bi^3+^ and VO_4_^3−^ produced the nanoparticles, which will assembly into a new across structure and branching crystal structure. The photocatalytic efficiency ranked as pH 5 > pH 3 > pH 7 > pH 9 > pH 1 > pH 11 > pH 13 > blank, which is in good agreement with XRD and SEM result. Perfect crystal in BiVO_4_ sample leads to high photocatalytic efficiency. *E. coli* Envelop was damaged in the presence of BiVO_4_ under visible light. The protrusion on the Envelop was diminished by the electrons and holes. ATR-FTIR results also suggested the intensity was weakened for the amide, phosphoric, –COO^−^ group and C-H even bond in LPS, peptidoglycan and periplasm molecules.

We herein reported morphology change of BiVO_4_in a wide range of pH under the same conditions by a facile preparation method. Due to the properties of VO_4_^3−^, the polymerization of vanadate with different degree resulted in the morphology change of BiVO_4_, which has not been reported. Under visible light, the damage of the cell was attributed to the free radical, generated by electrons and holes on BiVO_4_ surface. 

## Figures and Tables

**Table 1:** The length and thickness of BiVO_4_ prepared under different pH.

pH	Length (µm)	Thickness (µm)
0.5	20–30	2
1	10–20	1
3	1–9	0.5
5	0.8–1.0	0.1
7	0.1–1.0	0.05
9	0.05	0.01
11	1–3	0.01
13	5–10	0.01
